# Reactivating young muscle regenerative pathways in ageing through inhibition of the ‘gerozyme’ 15-hydroxyprostaglandin dehydrogenase

**DOI:** 10.1098/rstb.2026.0156

**Published:** 2026-11-12

**Authors:** Minas Nalbandian, Helen M. Blau

**Affiliations:** Microbiology and Immunology, Stanford University, Stanford, CA, USA

**Keywords:** 15-PGDH, gerozyme, sarcopenia, prostaglandin E2, muscle regeneration, ageing skeletal muscle

## Abstract

We are living longer, but the years lived free from chronic diseases and disabilities have not increased at the same rate. The loss of skeletal muscle function in late life constitutes a major barrier to achieving this goal. With age, muscle mass and strength progressively decline. When the loss of muscle function becomes pathological, it is clinically recognized as a syndrome, sarcopenia. The goal of our laboratory is to understand the cellular and molecular mechanisms that drive muscle ageing and sarcopenia and to identify therapeutic strategies that preserve or restore muscle function in older individuals. In this review, we discuss our discovery of the gerozyme 15-hydroxyprostaglandin dehydrogenase (15-PGDH), a molecular driver of ageing that impedes muscle tissue growth, maintenance and regeneration. 15-PGDH abundance and enzymatic activity increase with age, leading to degradation of prostaglandin E2 (PGE2), a lipid mediator with key roles in skeletal muscle regeneration and tissue homeostasis. By limiting PGE2 bioavailability, elevated 15-PGDH contributes to the impaired regenerative and adaptive capacity of aged muscle. Pharmacological inhibition of 15-PGDH restores PGE2 signalling and improves muscle mass, strength and regenerative capacity in old mice and constitutes a promising therapeutic strategy to counter sarcopenia and promote late-life muscle function.

This article is part of the theme issue ‘Developmental circuits in ageing’.

## 1. Introduction

Skeletal muscle is a critical determinant of mobility, metabolic health and longevity. After the age of 50, humans lose on average approximately 10% of muscle mass per decade, which progressively leads to reduced physical capacity and strength [1,2]. There are currently few effective strategies to maintain muscle function late in life. Exercise and nutritional interventions can slow the decline, but produce modest and inconsistent results, and no pharmacological intervention has been approved.

When age-related muscle loss progresses to the point of functional impairment, it is clinically recognized as sarcopenia. Sarcopenia is estimated to affect over 100 million people worldwide and is associated with frailty, loss of independence, hospitalization and increased mortality [3,4]. Despite decades of research, older adults at risk of losing their independence to sarcopenia have no treatment options.

We recently discovered that 15-hydroxyprostaglandin dehydrogenase (15-PGDH) drives the loss of muscle during ageing, leading us to define it as a ‘gerozyme’ [5]. 15-PGDH degrades prostaglandin E2 (PGE2), a lipid mediator traditionally associated with inflammation but now recognized as critical for skeletal muscle regeneration and homeostasis. In old muscle, we found that PGE2 levels decline, at least in part, because of increased 15-PGDH abundance and enzymatic activity. As discussed here, while phase I clinical trials have been completed and treatment with the 15-PGDH inhibitor appears safe [6], evidence of efficacy remains preclinical but striking, suggesting that inhibition of the 15-PGDH gerozyme represents a promising strategy to promote muscle anabolic growth and functional improvement in older individuals and patients with sarcopenia.

## 2. Muscle ageing: a global burden and unmet therapeutic need

Skeletal muscle is essential for voluntary movement, posture, joint stability, respiration, thermogenesis and whole-body metabolic homeostasis. With ageing, skeletal muscle undergoes progressive decline in mass, strength, power and regenerative capacity [7,8]. The clinical manifestation of severe age-associated muscle decline is sarcopenia. The definition of sarcopenia has evolved substantially in recent years. The European Working Group on Sarcopenia in Older People consensus defined sarcopenia in 2019 as a muscle disease rooted in adverse muscle changes across the life course and emphasized low muscle strength as the primary parameter. This represents a major shift in the definition of sarcopenia, from reduced muscle quantity alone towards reduced muscle quality, strength and performance [9]. Similarly, the Foundation for the National Institutes of Health Sarcopenia Project established clinically relevant cut-off values linking low lean mass and weakness to mobility impairment, reinforcing a shift from muscle mass alone towards function-based definitions [10]. Clinically, the major consequence is not only a loss of muscle mass, but also a loss of strength and functional capacity, which increases the risk of falls, fractures, institutionalization, loss of independence and mortality [11,12].

Sarcopenia is estimated to affect between 10% and approximately 30% of older individuals depending on diagnostic criteria, sex and geography [9,10,13], a range that reflects both epidemiological variation and the evolving consensus on how the condition should be defined and measured. The clinical importance of the diagnosis has been further reinforced by the creation of an International Classification of Diseases (ICD)-10-CM code for sarcopenia [14], which helped establish sarcopenia as a formally recognized disease entity rather than an inevitable consequence of ageing. This is particularly important because sarcopenia not only affects quality of life, but also imposes a substantial economic burden, and annual healthcare and hospitalization costs attributable to sarcopenia in the United States alone were estimated to exceed $40 billion in the year 2014 [3,4]. This societal burden highlights the need for therapeutic strategies that can counteract age-associated muscle decline.

Current management relies primarily on changes in exercise and nutrition, but their effects are modest and context-dependent. For example, a meta-analysis of randomized and non-randomized clinical trials in adults aged 50 years and older found that resistance exercise improves grip strength, leg press strength and gait speed in older adults [15], and the European Society for Clinical Nutrition and Metabolism guidelines recommend daily physical activity combined with adequate protein intake as the optimal approach for older adults [16]. However, these interventions often fall short in the populations most affected by sarcopenia, including frail, post-hospitalized and sarcopenic obese individuals, for whom structured exercise may be difficult to implement and insufficient to restore anabolic responsiveness. For instance, the DO-HEALTH trial, which enrolled more than 2000 community-dwelling adults aged 70 years and older, found that vitamin D3, omega-3 supplementation and home-based strength exercise over three years did not significantly improve lower-extremity function, grip strength or muscle mass [17]. Thus, even under relatively favourable conditions, lifestyle-based interventions may be insufficient to restore muscle function to a meaningful extent in ageing. These findings underscore the need for pharmacological strategies that directly enhance the capacity of aged muscles to respond to anabolic stimuli.

## 3. Cellular and molecular hallmarks of old skeletal muscle

At the tissue level, skeletal muscle ageing reflects a coordinated decline across the contractile, neural, vascular, stromal, inflammatory and stem cell compartments. In human muscle biopsies collected across a wide age range, ageing reduces both total fibre number and fibre size [18], with the loss driven predominantly by atrophy of fast type II myofibres, whereas type I fibres are preserved [19]. A recent study extended this observation by showing that, in human muscle, type II fibre deterioration in ageing is accompanied by increased denervation and reduced vascular and satellite cell support around type II fibres [20]. In agreement, a recent multimodal single-cell and single-nucleus atlas of human skeletal muscle showed that ageing reduces type II myonuclei, particularly within the type IIX compartment, and is associated with the emergence of abnormal myonuclear states enriched for denervation-, stress-, inflammatory- and proteolysis-related programmes [21]. This selective type II fibre vulnerability is critical, because these fibres are required for rapid, high-force contractions that support power, balance and fall prevention. Neuromuscular deterioration compounds this vulnerability—in old muscle, denervated fibres can persist even in the presence of nearby motor axons, indicating that reinnervation becomes inefficient with age [22], and, as we recently reported [23], this impairment progresses in severity across the clinical stages of sarcopenia. With ageing, myofibres do not simply shrink, but instead acquire transcriptional states consistent with chronic stress, failed repair and impaired neuromuscular maintenance.

In parallel, muscle stem cells (MuSCs), also known as satellite cells, undergo functional decline with ageing. MuSCs, originally identified as mononuclear cells positioned at the periphery of skeletal muscle fibres [24], contribute to muscle maintenance and growth [25]. We identified oncostatin M as a secreted niche factor that induces MuSC quiescence, demonstrating that extrinsic signals actively preserve the resting stem cell state rather than merely activating MuSCs during repair [26]. With ageing, MuSCs undergo cell-intrinsic changes, lose quiescence and enter a senescent-like state, which limits their ability to re-enter the cell cycle and support regeneration [27]. Ageing also alters MuSC fate through changes in extrinsic signals from the niche—increased Wnt signalling in old muscle was shown to bias MuSCs away from myogenesis and towards fibrogenesis [28]. Indeed, the MuSC population has been reported to decline in older women and men, by 24 and 37%, respectively [29]. More recently, human single-nuclei profiling showed that aged MuSCs are depleted and shift towards an early-primed state, consistent with premature activation and exhaustion of the stem cell pool [21]. Fewer stem cells with reduced proliferative capacity signal depletion of the regenerative reserve and an inability to meet the need when muscle is damaged or stressed.

The niche that supports MuSCs also deteriorates with age. For example, in human muscle, capillary density and microvascular function have been shown to decline with ageing, suggesting that vascular rarefaction may limit nutrient delivery and oxygen exchange, which can impact remodelling capacity [30]. At single-cell resolution, ageing is also associated with reduced capillary endothelial cells and pericytes. Moreover, existing vascular cells become less functional owing to increased inflammation and transforming growth factor-beta (TGF-β) signalling, disrupting endothelial integrity [21]. The extracellular matrix (ECM) also changes with age—old muscle matrix has been shown to drive a fibrogenic conversion of MuSCs, directly linking ECM remodelling to impaired regenerative fate decisions [31]. Fibro/adipogenic progenitors typically support myogenesis after acute injury, but related mesenchymal progenitors are known to cause fibrosis or ectopic fat accumulation under pathological conditions, showing how stromal populations can shift from repair-supportive to maladaptive states [32–34]. This stromal shift is reflected by an expansion of fibroblast-like cells and an increase in extracellular matrix, interleukin-6 (IL-6) and TGF-β signalling programmes in fibro-adipogenic populations. Immune signalling follows a similar principle—immune cells are required for muscle growth and regeneration, but in ageing, inflammatory signalling becomes chronic, which can disrupt the timing and resolution of repair [35,36]. Accordingly, ageing increases immune-cell infiltration and remodels immune-cell states, including increased myeloid and mast-cell populations and enrichment of pro-inflammatory signalling programmes [21]. Thus, vascular decline, ECM remodelling and chronic low-grade inflammation converge to create a niche that not only fails to support regeneration but actively biases MuSC fate away from myogenesis.

Work from our laboratory has revealed that aged MuSC dysfunction involves cell-intrinsic defects that persist even when niche conditions are improved. We found that aged MuSCs have a markedly reduced frequency of functionally competent stem cells [37]. Using transplantation of fluorescence-activated cell sorting (FACS)-purified young or aged MuSCs into injured recipient muscle, we found that aged MuSCs displayed impaired regenerative function, even when placed in a permissive young microenvironment. Limiting-dilution transplantation experiments, in which progressively smaller numbers of MuSCs were transplanted to estimate the frequency of cells capable of successful engraftment, revealed that aged MuSCs contain an approximately two-thirds lower frequency of functional stem cells than young MuSCs, demonstrating that the defect is cell-autonomous.

Mechanistically, this loss of function was associated with elevated p38-α/ β mitogen-activated protein kinase (MAPK) signalling [37]; however, inhibiting the kinase did not suffice to increase engraftment. Our lab had previously shown that culturing MuSCs on soft hydrogel substrates matching the stiffness of young muscle maintains their potential for self-renewal, which is rapidly lost if plated on material with a stiffness of tissue culture plastic [38]. By combining transient p38- α/β inhibitor treatment with a muscle-mimetic hydrogel substrate, aged MuSC regenerative function was restored [37]. These experiments showed that biochemical and biophysical signals can synergize to overcome the loss of stemness with ageing. We further discovered that MuSCs retain a mechanical memory of prior substrate stiffness, i.e. that exposure to the rigid environment of old muscle has lasting effects on YAP/TAZ-mediated mechanosignalling and stem cell function, even after stiffness is reduced [39]. These studies highlight the negative impact of fibrosis on stem cells, a condition that increases in many tissues owing to disease or ageing.

We did not have a way to isolate dysfunctional aged stem cells until single-cell mass cytometry revealed that CD47 upregulation marks a dysfunctional aged MuSC population, a finding that enabled their isolation by FACS and targeting of CD47 as a way to enhance regeneration [40]. This work also revealed that the CD47-high aged MuSC state is driven, in part, by altered U1 small nuclear RNA (snRNA)-dependent CD47 RNA processing and impaired cyclic adenosine monophosphate (cAMP) signalling, providing mechanistic insights into how this dysfunctional population arises [40]. In further studies, we revealed the critical role of primary cilia in stem cell function and that these organelles are lost from MuSCs with ageing, impairing Sonic Hedgehog signalling and regenerative capacity [41]. Restoring the Hedgehog pathway with a Smoothened agonist rescued aged MuSC function. Together, these studies establish that aged MuSC dysfunction arises from the convergence of intrinsic stress signalling pathways, mechanobiological reprogramming and cell-surface alterations—not simply from loss of niche factors.

At the molecular level, old muscle shows defects in energy metabolism, proteostasis and anabolic responses. In human skeletal muscle, ageing is associated with reduced mitochondrial oxidative capacity and mitochondrial DNA abundance and increased oxidative damage, linking mitochondrial dysfunction to age-associated muscle decline [42]. In physically active older adults with muscle atrophy, mitochondria show increased sensitivity to permeability transition pore opening and nuclear translocation of endonuclease G, a mitochondria-resident nuclease released during apoptotic stress, indicating that mitochondrial dysfunction persists even in old muscle that remains physically active [43]. Protein turnover is also altered—muscle wasting involves activation of ubiquitin and autophagy–lysosome systems, which mediate protein degradation during atrophy [44]. In line with these findings, aged human myonuclei show downregulation of metabolic and sarcomeric genes together with enrichment of autophagy, ubiquitin–proteasome, forkhead box O (FOXO), inflammatory and stress-response pathways [21].

Older individuals often find it hard to build skeletal muscle owing to ‘anabolic resistance’, which is defined as a reduced ability to increase muscle protein synthesis in response to anabolic stimuli, such as amino acids and resistance exercise [45]. Older adults exhibit reduced muscle protein synthesis after essential amino acid ingestion, accompanied by impaired activation of anabolic signalling pathways, supporting the concept of ‘amino acid resistance’ in ageing muscle [46,47]. This is reflected in muscle force and mass. After approximately six weeks of resistance training, young individuals exhibit an approximately 20% increase in strength, whereas older individuals typically achieve only approximately 5% gain, reflecting a markedly blunted hypertrophic response to the same mechanical loading with ageing [48]. In part, this appears to reflect altered kinetics and reduced efficiency of protein synthesis; when essential amino acid supplementation was combined with resistance training, muscle protein synthesis increased in response to anabolic stimulation, but the response was delayed in older men [49]. At the level of signalling, the aged human muscle niche also shows dysregulated growth-factor communication, including altered activin, insulin-like growth factor (IGF), bone morphogenetic protein (BMP), Notch and Wnt pathways, providing an additional molecular basis for impaired anabolic and regenerative responses [21,50]. Consequently, in old muscle, the problem is not only tissue loss but also a diminished capacity to respond appropriately to anabolic, mechanical and regenerative cues.

## 4. Prostaglandin E2 as an essential lipid mediator of muscle regeneration and muscle homeostasis

Healthy skeletal muscle is characterized by a remarkable regenerative capacity [51], largely enabled by a resident muscle-specific stem cell population termed MuSCs or satellite cells [24]. Under homeostatic conditions, most MuSCs reside beneath the basal lamina in a quiescent state. Following injury, MuSCs spring into action—they become activated, undergo symmetric or asymmetric divisions to expand the pool and generate committed myogenic progeny and then differentiate into myoblasts that fuse with damaged fibres to rebuild muscle [52–54]. This process requires precise coordination with infiltrating immune cells, stromal cells, endothelial cells and the extracellular matrix.

Margaret Buckingham provided early evidence of MuSCs using FACS, showing that a pure population of satellite cells, isolated from a Pax3-GFP mouse transgenic model, contributed very efficiently to skeletal muscle regeneration and also self-renewed [55]. Work from our laboratory established definitively that individual MuSCs are bona fide stem cells. Sacco *et al.* [56] showed that the progeny of a single transplanted MuSC can extensively proliferate, contribute to regenerated myofibres and give rise to Pax7⁺ cells that can be reisolated—demonstrating both self-renewal and differentiation at the clonal level. This finding was enabled by bioluminescence imaging of a constitutively expressed luciferase construct stably expressed in each stem cell of a donor transgenic mouse model that could be tracked non-invasively over time [56].

Muscle regeneration is governed by a temporally coordinated inflammatory programme during which monocytes and macrophages are recruited to clear damaged tissue and stimulate MuSC proliferation and differentiation to replenish lost myofibres, while cytokine signalling pathways, such as IL-6/STAT3 and IL-4/IL-13, coordinate MuSC and fibro-adipogenic progenitor responses [57–61]. The subsequent resolution phase is mediated by specialized pro-resolving lipid mediators, including resolvins, which constrain inflammation and facilitate myofibre growth and functional recovery [62,63]. Among the niche-derived signals that coordinate this response, PGE2 has emerged as a central lipid mediator linking the early inflammatory phase of injury to MuSC activation and repair [64].

PGE2 was first shown to play a role in controlling stem cells in haematopoietic stem cells (HSCs). A chemical genetic screen using zebrafish embryos identified PGE2 as a regulator of HSC homeostasis [65]. This finding was extended to human cells when *ex vivo* treatment of cord blood CD34^+^ HSCs with a more stable PGE2 analogue, 16,16-dimethyl-PGE2 (dmPGE2), was shown to enhance their engraftment capacity following transplantation [66].

In skeletal muscle, early regeneration is characterized by a transient pro-inflammatory microenvironment that promotes MuSC activation and proliferation [33,36,67–69]. We have shown that PGE2 is an essential mediator of this response, required for MuSC proliferation and survival during regeneration [64,68,70–73]. PGE2 is derived from arachidonic acid via the cyclooxygenase pathway [74,75] (figure 1), is rapidly produced in response to tissue injury, and acts locally through four G-protein-coupled receptors, EP1–EP4. Following activation, MuSCs upregulate EP4, the predominant PGE2 receptor on MuSCs and myofibres that mediates muscle repair [64,70]. Upon MuSC-specific deletion of EP4, or pharmacological blockade of PGE2 synthesis with a non-steroidal anti-inflammatory drug (NSAID), the ability of MuSCs to proliferate and meet the need for muscle repair after damage was reduced and muscle force was not recovered after injury [64]. These experiments changed the interpretation of PGE2 in injured muscle—rather than being merely an inflammatory by-product, PGE2 is functionally required for MuSC expansion, myofibre repair and recovery of strength. Single-cell tracking of MuSCs from young and old mice revealed that old MuSCs exhibit a specific defect in proliferation and survival—the cellular basis for the two-thirds reduction in regenerative potential shown by Cosgrove *et al.* [37]. The striking effects of PGE2 on old MuSCs were revealed by time-lapse microscopy experiments charting the fate of single old MuSCs. Upon exposure to PGE2 in hydrogel microwells mimicking the stiffness of young muscle, the necroptosis underlying mitotic catastrophe was overcome and their proliferative capacity was restored [70].

**Figure 1.**
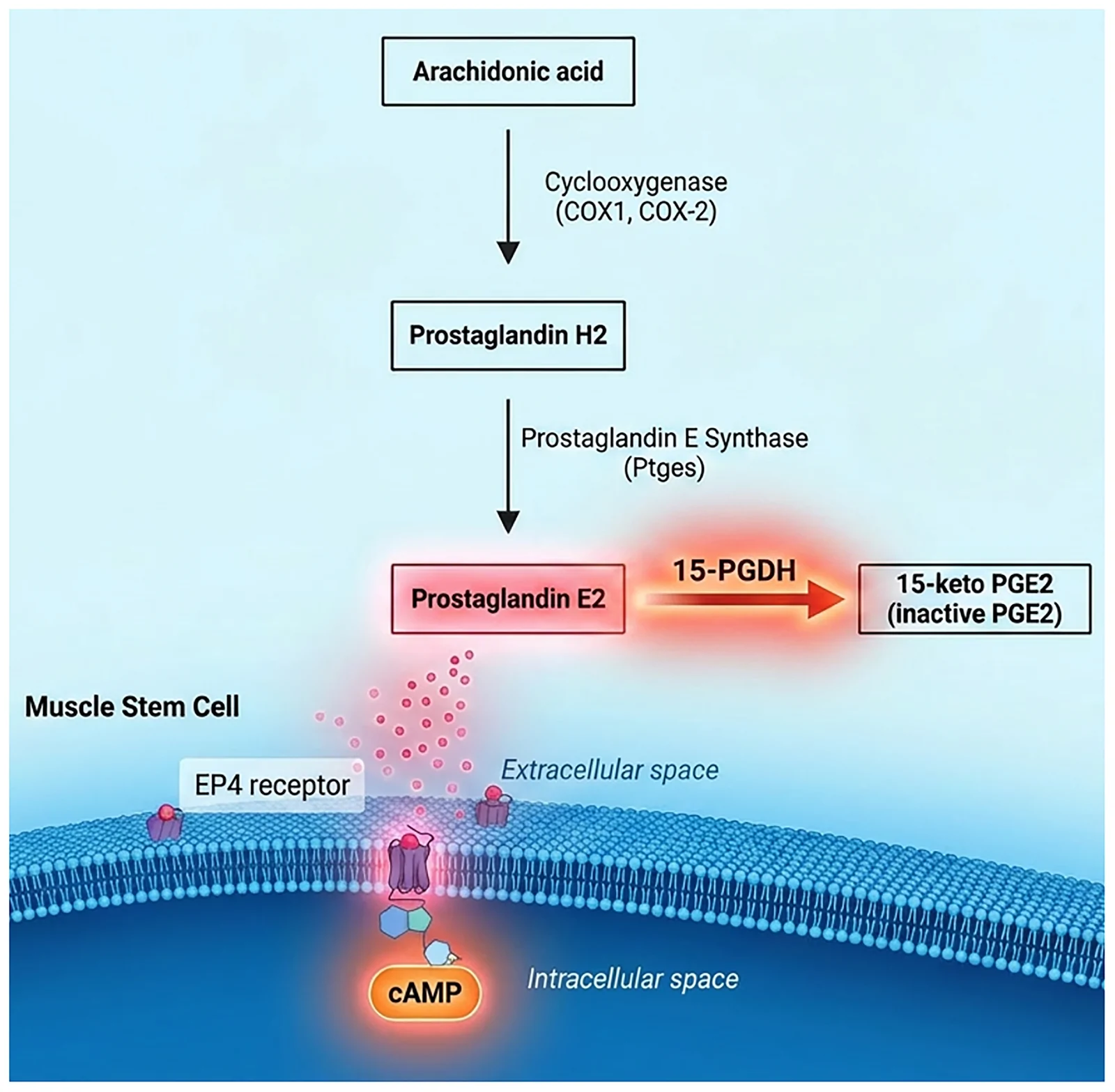
The 15-PGDH/PGE2/EP4 signalling axis in muscle stem cells. Arachidonic acid is converted by cyclooxygenase enzymes COX-1 and COX-2 into prostaglandin H2, which is subsequently converted by prostaglandin E synthases into prostaglandin E2 (PGE2). Extracellular PGE2 can bind the EP4 receptor on MuSCs, activating intracellular cyclic adenosine monophosphate (cAMP) signalling. PGE2 bioavailability is limited by 15-hydroxyprostaglandin dehydrogenase (15-PGDH), which oxidizes PGE2 to the inactive metabolite 15-keto-PGE2.

These cell-based findings are supported by loss-of-function studies *in vivo.* In mice with MuSC-specific deletion of EP4, regeneration after injury was reduced, as evidenced by smaller regenerating myofibres and reduced recovery of muscle force [64]. Similarly, pharmacological blockade of PGE2 production in wild-type mice using NSAIDs, which inhibit the COX-1 and COX-2 enzymes required for prostaglandin synthesis, impaired muscle regeneration [64]. In the absence of this signalling pathway, in contrast to controls, force fell and was not restored after injury. Mechanistically, PGE2–EP4 activates cAMP–protein kinase A (PKA) downstream signalling cascades that drive MuSC proliferation and regenerative output [64,70]. These genetic and pharmacological experiments show that PGE2 is not simply a by-product of inflammation after injury, but is instead essential for efficient MuSC expansion, myofibre regeneration and recovery of muscle strength.

Beyond acute injury, PGE2 signalling also contributes to long-term muscle maintenance. It has become increasingly apparent that MuSCs contribute not only to acute repair, but also to myofibre homeostasis throughout adult life [76–78]. Strikingly, mice with MuSC-specific deletions of EP4 developed smaller muscles and exhibited reduced strength by 14 months of age—even in the absence of any experimentally induced injury [70]. These findings indicate that PGE2–EP4 signalling in MuSCs may contribute to the preservation of muscle mass and function during ageing, by maintaining MuSC regenerative competence and supporting the repair of low-grade damage that accumulates over time and cellular turnover.

Human and mouse exercise studies are in agreement and indicate that COX-derived prostaglandins participate in skeletal muscle adaptation to mechanical loading. In young men, eccentric resistance exercise increased intramuscular PGE2, while NSAIDs suppressed this post-exercise increase [79], in support of the idea that prostaglandin production is part of the early biochemical response to contractile stress. The same group reported that over-the-counter doses of ibuprofen or acetaminophen suppressed the post-exercise increase in muscle protein synthesis [80] and ibuprofen also blunted early translational signalling—including mitogen-activated protein kinase kinase (MEK)–extracellular signal-regulated kinase (ERK) activation—after resistance exercise [81]. Prior acetaminophen consumption similarly suppressed the early mTORC1 response to acute resistance exercise [82]. Mouse models of overload-induced hypertrophy also showed that pharmacological COX-2 inhibition almost completely negated the hypertrophy response of skeletal muscle [83]. Beyond acute signalling responses, in young adults undergoing eight weeks of resistance training, daily high-dose ibuprofen attenuated increases in muscle volume and strength compared with low-dose aspirin [84], indicating that COX-2 activity is required for muscle growth in response to increased loading.

Prostaglandin-dependent effects are, in part, mediated through MuSCs. NSAID injection into human skeletal muscle after unaccustomed eccentric exercise inhibited satellite cell proliferation, indicating that local cyclooxygenase activity contributes to the myogenic precursor response after loading [85]. NSAIDs administered orally attenuated the exercise-induced increase in satellite cell number [86]. These human and mouse data establish prostaglandin signalling as a conserved coordinator of anabolic adaptation to mechanical loading and exercise.

In a recent multiomic study, we analysed the direct effects of PGE2 signalling in old muscle [70]. Old MuSCs showed reduced PGE2–EP4 signalling, and single-cell tracking confirmed defects in survival and cell-cycle re-entry. Pseudotime analysis of single-cell RNA sequencing revealed that, after injury, MuSCs enter one of two trajectories: a cycling trajectory supporting effective regeneration or a premature commitment trajectory that bypasses the transient amplifying state. Old MuSCs were biased towards premature commitment, explaining why they are unable to meet the proliferative demands required for effective regeneration. Concordantly, genes associated with PGE2–EP4/CREB signalling were enriched along the cycling trajectory. PGE2 treatment of aged MuSCs restored EP4/CREB signalling, suppressed AP-1-associated programmes, increased expression of cycling-trajectory genes and improved MuSC proliferation and survival [70]. Together, these findings support a model in which age-associated loss of PGE2–EP4 signalling contributes to altered MuSC fate progression, whereas restoration of this pathway promotes a more regenerative, proliferative state. At the chromatin level, PGE2 partially reversed age-associated changes in accessibility—increasing accessibility at cAMP response element (CRE) and E-box motifs and reducing AP-1 binding [70]. Functionally, short-term PGE2 exposure of old MuSCs had lasting effects. Transplanted old MuSCs pretreated with PGE2 showed enhanced engraftment and regenerative potential *in vivo*, with their progeny retaining enhanced proliferative capacity and viability [70]. These data show that old MuSCs are not irreversibly defective. Part of their dysfunction reflects loss of a PGE2-responsive state that can be readily restored.

## 5. 15-Hydroxyprostaglandin dehydrogenase: a gerozyme and ageing-induced brake on prostaglandin E2 signalling

Given the central role of PGE2 in MuSC function, muscle regeneration and muscle maintenance, restoring PGE2 signalling in old muscle has emerged as a promising strategy to counter age-associated muscle decline. However, direct administration of PGE2 poses several limitations. PGE2 is rapidly metabolized and has a short biological half-life, requiring sustained local availability to maintain its activity. In addition, systemic bolus administration could produce unwanted effects on cell types other than skeletal muscle; for instance, vascular effects such as vasodilation that could lead to syncope [87]. These limitations suggest that, instead of systemic PGE2 delivery, a strategy designed to increase endogenous PGE2 signalling within tissues may be preferable.

The identification of 15-PGDH as an age-induced PGE2-degrading enzyme provided a direct mechanism by which reduced PGE2 signalling could occur in old muscle. Our laboratory has shown that 15-PGDH expression and activity increase with age across multiple tissues and that, in old skeletal muscle, this elevation is accompanied by reduced PGE2 levels, establishing that ageing imposes an enzymatic constraint on a central regenerative signalling pathway [5].

This suggests a different therapeutic strategy—rather than administering PGE2 directly, inhibiting its degradation. The feasibility of this approach was established by earlier work from Zhang *et al*. [88], who identified SW033291 as a potent small-molecule inhibitor of 15-PGDH and showed that pharmacological 15-PGDH inhibition increases tissue PGE2 levels and enhances tissue repair in several regenerative settings, including bone marrow, colon and liver [88,89]. Unlike exogenous PGE2 administration, inhibition of 15-PGDH increases PGE2 by preventing its enzymatic degradation, thereby amplifying endogenous PGE2 signalling in tissues in which the pathway is active or 15-PGDH is elevated. More broadly, these studies established 15-PGDH as a druggable negative regulator of tissue repair, providing the foundation for testing whether age-associated increases in 15-PGDH similarly constrain regenerative pathways in old skeletal muscle.

In old mice, one month of treatment with a 15-PGDH inhibitor was sufficient to increase muscle mass, fibre size, mitochondrial function, force and treadmill performance [5,90]. At the molecular level, 15-PGDH inhibition was associated with increased expression of mitochondrial electron transport chain genes and reduced expression of pathways linked to TGF- β signalling, protein degradation and muscle atrophy, including atrogenes and myostatin-associated programmes [5]. Conversely, ectopic overexpression of 15-PGDH in young mouse muscles caused muscle atrophy and weakness, producing features of premature muscle ageing in young animals [5]. These loss- and gain-of-function experiments establish 15-PGDH as a ‘gerozyme’—an enzyme that increases with age, drives functional decline when elevated and promotes rejuvenation when inhibited (figure 2).

**Figure 2.**
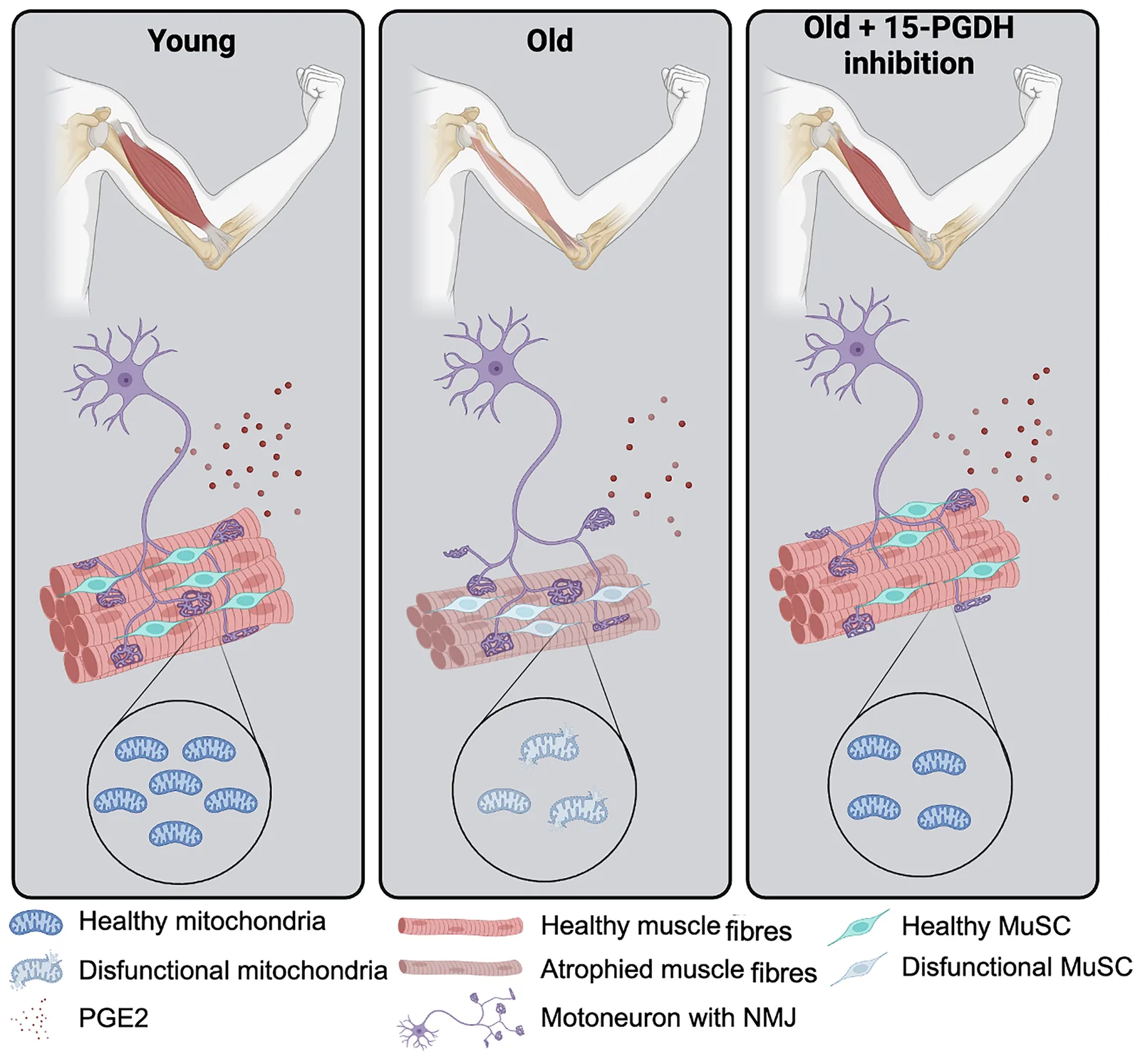
15-Hydroxyprostaglandin dehydrogenase (15-PGDH) inhibition restores features of youthful muscle signalling and function in aged skeletal muscle. Schematic representation of age-associated changes in skeletal muscle and their modulation by 15-PGDH inhibition. Young muscle is characterized by preserved muscle structure, robust neuromuscular input, higher prostaglandin E2 (PGE2) availability and maintained mitochondrial content. With ageing, skeletal muscle undergoes structural deterioration, reduced regenerative and neuromuscular support, diminished PGE2 signalling and mitochondrial decline. Inhibition of 15-PGDH increases PGE2 bioavailability and restores a more youthful muscle state, improving muscle integrity, mitochondrial abundance and functional capacity in aged muscle. MuSC, muscle stem cell; NMJ, neuromuscular junction.

The effects of 15-PGDH inhibition are not limited to myofibre size and contractile function. In a subsequent study, we showed that pharmacological inhibition of 15-PGDH promotes regeneration of motor axons and formation of neuromuscular synapses after acute and chronic denervation [90]. After acute nerve crush injury, 15-PGDH inhibition accelerated recovery of muscle force, whereas in old mice, the inhibitor increased motor neuron viability, restored neuromuscular junctions and improved muscle function. Mechanistically, the study linked these effects to increased PGE2 signalling and CREB activation in motor neurons, suggesting that 15-PGDH inhibition can restore nerve–muscle connectivity as well as myofibre function [90]. To our knowledge, 15-PGDH inhibition is the first pharmacological intervention shown to restore neuromuscular innervation and improve muscle function in aged or chronically denervated muscle, with voluntary exercise being the only non-pharmacological intervention reported to produce similar benefits in old mice [91]. If this finding in mice translates to humans, it could be particularly impactful for sarcopenia, where preferential type II fibre atrophy, denervation and impaired neuromuscular junction maintenance are major contributors to loss of strength and power.

Thus, 15-PGDH sits upstream of several age-sensitive processes in muscle, including MuSC function, mitochondrial activity, myofibre maintenance and neuromuscular repair. By limiting PGE2 availability, increased 15-PGDH appears to suppress a repair pathway that remains responsive in old tissue. Because 15-PGDH can oxidize prostaglandins and related hydroxylated lipid mediators in addition to PGE2, we cannot rule out a role for additional prostanoids or bioactive eicosanoids in the observed effects of 15-PGDH inhibition. Nevertheless, current genetic gain- and loss-of-function experiments, pharmacological or antibody-mediated inhibition of PGE2 signalling and experiments in which the stable analogue 16,16-dimethyl PGE2 (dmPGE2) was delivered directly to mice provide strong support for restoration of PGE2 signalling as a major mechanism by which 15-PGDH inhibition improves muscle mass and function. Taken together, this makes 15-PGDH inhibition an attractive therapeutic strategy for sarcopenia.

## 6. Translational outlook: clinical development and future opportunities

A burning question is whether the preclinical effects can be reproduced in humans. MF-300, an orally administered 15-PGDH inhibitor developed for sarcopenia, has completed phase 1 testing in healthy volunteers and older adults [6]. Publicly reported phase 1 data indicate that MF-300 was generally well tolerated across evaluated dose ranges, with no dose-limiting toxicities, pharmacokinetics compatible with once-daily dosing and pharmacodynamic evidence of target engagement based on dose-related changes in PGE2 and its metabolites. These data support the feasibility of increasing endogenous PGE2 signalling through inhibition of its degrading enzyme rather than through direct PGE2 administration. A phase 2b trial in age-related sarcopenia has now been announced. The critical test will be whether these effects translate into measurable improvements in strength, physical performance or recovery capacity in sarcopenic older adults.

Additional preclinical contexts may broaden the relevance of the pathway. One emerging area is pharmacologically induced weight loss. Glucagon-like peptide-1 (GLP-1) receptor agonists are highly effective anti-obesity therapies, but rapid or substantial weight loss can be accompanied by loss of lean mass, raising the question of how to preserve muscle quality and regenerative capacity during treatment. We recently reported that 15-PGDH inhibition counteracts muscle regenerative deficits associated with GLP-1 receptor agonist-induced weight loss, supporting the possibility that 15-PGDH inhibition could help preserve repair capacity in settings where weight loss imposes stress on skeletal muscle [92].

A second application is the potential interaction between 15-PGDH inhibition and exercise. Exercise remains the most effective non-pharmacological intervention for preserving muscle health, but old muscle often shows blunted anabolic and regenerative responses. Since prostaglandin signalling contributes to muscle adaptation after mechanical stress and since 15-PGDH inhibition restores endogenous PGE2 signalling in old muscle, an important future direction is to determine whether 15-PGDH inhibition can enhance the response to exercise, resistance training or overload in old animals and ultimately in older adults. This would position 15-PGDH inhibition not as a substitute for exercise, but as a way to overcome anabolic resistance and improve the responsiveness of old muscle to mechanical stimuli.

Finally, the relevance of 15-PGDH inhibition may extend beyond skeletal muscle. 15-PGDH regulates PGE2 availability in multiple tissues and pharmacological inhibition of 15-PGDH has been explored in regenerative contexts involving cartilage [93], bone [94], haematopoiesis [95], liver [96,97] and fibrotic disease settings [98,99]. If this pattern holds across tissues, 15-PGDH inhibition may qualify as a geroprotective strategy—one that targets not a single organ but the enzymatic brake that ageing imposes on repair.

## Data Availability

This article has no additional data.

## Theme

One contribution of 21 to a theme issue ‘Developmental circuits in ageing’.
